# Prevalence of *FLT3* gene mutation and its expression in Brazilian pediatric B-ALL patients: clinical implications

**DOI:** 10.3389/fped.2024.1505060

**Published:** 2024-12-06

**Authors:** Estefânia Rodrigues Biojone, Bruna Cândido Guido, Larissa Lemos Mendanha Cavalcante, Agenor de Castro Moreira dos Santos Júnior, Robéria Mendonça de Pontes, Felipe Magalhães Furtado, José Carlos Córdoba, Isis Maria Quezado Magalhães, Diêgo Madureira de Oliveira, Ricardo Camargo

**Affiliations:** ^1^ Oncology and Hematology Division, Children’s Hospital of Brasília, Brasília, Brazil; ^2^ Laboratory of Translational Research, Children’s Hospital of Brasília, Brasília, Brazil; ^3^ Department of Hematology, Sabin Diagnóstico e Saúde, Brasília, Brazil; ^4^ Multidisciplinary Health Laboratory, Faculty of Health Sciences and Technology, University of Brasília, Brasília, Brazil

**Keywords:** precursor B-cell lymphoblastic leukemia-lymphoma, precision medicine, molecular biology, tumor biomarkers, child health

## Abstract

**Introduction:**

There is consistent evidence that *FLT3* may be a driver gene in B-ALL and that selected cases may benefit from the use of FLT3 inhibitors. Our study was conducted to evaluate the frequency and types of FLT3 mutations in pediatric patients with B-ALL, the relative expression of this gene, and their influence on clinical evolution.

**Methods:**

We evaluated 156 children with B-ALL treated between July 2018 and September 2023. Screening for FLT3 mutations was performed using RFLP and fragment analysis, while FLT3 expression was assessed by qPCR.

**Results:**

*FLT3*-TKD and/or *FLT3*-JM-INDEL mutations were found in 8 patients (5.1%). We did not identify any ITD-type mutations. None of the patients with identified *FLT3* mutations presented recurrent rearrangements in B-ALL or alterations in the *IKZF1*, *PAX5*, or *ERG* genes, suggesting that *FLT3* mutation may serve as the driving mechanism for leukemia in these cases. Two (2/8) patients with *FLT3* mutations experienced disease relapse. Although we did not observe *FLT3* overexpression among patients with *FLT3* mutations, *FLT3* expression levels were higher in these patients compared to WT patients. Four *FLT3*-WT patients presented *FLT3* overexpression, defined as RQ > 10. *FLT3* mutations or overexpression were not associated with relapses or survival rates.

**Discussion:**

Our findings do not support the inclusion of *FLT3* as a routine marker in the risk stratification of B-ALL patients; nevertheless, FLT3 alterations may be relevant for guiding personalized treatment approaches in specific clinical contexts.

## 1 Introduction

B-cell Acute Lymphoblastic Leukemia (B-ALL) is the most common cancer in the pediatric population, accounting for approximately 25% of malignant neoplasms in patients up to 18 years of age (1, 2). Despite significant improvements in survival rates, relapsed or refractory disease remains a frequent cause of death among B-ALL patients (3). Currently, the assessment of leukemic cells through molecular biology techniques and genetic evaluation, including next-generation sequencing (NGS), enables the identification of over thirty ALL subtypes characterized by specific gene expression profiles or biological markers (4). This characterization not only enhances diagnostic accuracy but also provides opportunities for treatment optimization, either by adjusting chemotherapy intensity or by introducing targeted therapies (5).

The *FLT3* (FMS-like tyrosine kinase 3) gene, located on chromosome 13q12, encodes a type III receptor tyrosine kinase predominantly expressed in the bone marrow, particularly in hematopoietic precursor cells. The protein consists of four distinct regions: an extracellular domain, a transmembrane region, a juxtamembrane region, and an intracellular portion containing a tyrosine kinase domain. Upon binding to the *FLT3* ligand (FL), the receptor is activated through dimerization and autophosphorylation, initiating a cascade of signaling pathways, including PI3K/AKT, RAS/MAPK, and STAT5 (6, 7). This signaling promotes cell proliferation and inhibits apoptosis. The *FLT3* gene plays a critical role in the survival, proliferation, and differentiation of hematopoietic cells across both myeloid and lymphoid lineages (6, 8–11).

Mutations in *FLT3*, occurring either in the juxtamembrane domain or within the tyrosine kinase domain, lead to constitutive activation of *FLT3* and are associated with leukemogenesis (12–20). There are four main types of activating mutations in the *FLT3* gene: internal tandem duplications in the juxtamembrane domain (*FLT3*-ITD), in-frame insertions or deletions in the juxtamembrane domain (*FLT3*-JM-INDEL), point mutations in the juxtamembrane domain (*FLT3* JM-PM), and mutations in the tyrosine kinase domain (*FLT3*-TKD) (21).

*FLT3*-activating mutations are commonly found in Acute Myeloid Leukemias (AML), accounting for approximately one-third of adult AML cases and 10%–15% of pediatric AML cases (19). Among AML patients, *FLT3*-ITD is a recurrent driver mutation (present in about 25% of all AML cases) and is associated with higher relapse rates and reduced overall survival (12–26). In contrast, TKD domain mutations are likely secondary events with uncertain prognostic impact (22, 23). FLT3 inhibitor drugs are approved for AML patients with *FLT3* mutations, and their use has been associated with improved overall survival and event-free survival rates (26–30).

In B-ALL, *FLT3* mutations are less common, reported in 0.2%–12.5% of cases when evaluated by conventional techniques (RFLP and fragment analysis) (31–36) and in up to 25% when investigated using NGS (20). The prognostic impact of these alterations remains poorly defined (20, 30–36). ITD-type mutations are rare in this context, and recent studies have described in-frame indels in the juxtamembrane domain as the most common type of *FLT3* genetic variant in patients with B-ALL (21).

*FLT3* overexpression, in addition to its activating mutations, has been documented in B-ALL, particularly in specific subtypes such as r-*KMT2A*, r-*ZNF384*, and high hyperdiploid ALL (20, 37–41). The mechanisms contributing to elevated *FLT3* expression in certain B-ALL subtypes are not yet fully understood. Epigenetic modifications, such as enhancer hijacking due to deletions at 13q12.2, have been linked to higher expression levels in hyperdiploid patients and in cases of relapse (42). Although the surface expression of the *FLT3* receptor does not correlate with *FLT3* transcript levels, total cellular *FLT3* protein levels generally reflect transcript levels. Overexpressed wild-type *FLT3* proteins have been observed to undergo tyrosine phosphorylation (43). Some studies have associated elevated *FLT3* expression with poorer outcomes in B-ALL (20, 36–40, 44). Encouragingly, the autophosphorylation of wild-type *FLT3* induced by its overexpression was shown to be inhibited by a potent *FLT3* kinase inhibitor, with sensitivity comparable to that observed in mutant forms (43, 45). Additionally, evidence suggests therapeutic responsiveness to FLT3 inhibitors in certain subsets of relapsed B-ALL patients exhibiting *FLT3* overexpression (46–48). Nevertheless, few studies have investigated therapeutic strategies involving FLT3 inhibitors specifically for B-ALL (41, 49).

Given the current uncertainty regarding the prognostic value of *FLT3* alterations in pediatric B-ALL and considering recent reports of the therapeutic efficacy of FLT3 inhibitors in patients with relapsed B-cell ALL (46–48), it is essential to deepen our understanding in this field of research. The objective of this study was to identify *FLT3* alterations in patients with B-ALL and to correlate these alterations with their clinical course to clarify whether *FLT3* is a molecular marker of clinical relevance in children with B-ALL.

## 2 Materials and methods

### 2.1 Study design

This is a retrospective, descriptive clinical study involving pediatric patients diagnosed with B-ALL at a public pediatric oncology referral hospital in Brasília, Brazil. A convenience sampling was performed, including patients admitted between July 2018 and September 2023. Data collected comprised clinical characteristics (age, gender, white blood cell count at diagnosis, and the presence of Central Nervous System—CNS—infiltration), biological characterization (cytogenetic alterations, recurrent rearrangements, and mutations in *IKZF1*, *PAX5*, and *ERG*), and treatment response measured by Minimal Residual Disease (MRD). Outcomes were categorized as remission, relapse or dead in remission.

### 2.2 Study population and treatment

Patients aged 1–18 years with a primary diagnosis of B-ALL and no prior treatment were included. Between July 2018 and September 2022, treatment was based on the BFM ALLIC 2009 protocol, locally adapted for B-ALL management at our institution. Since September 2022, the GBTLI 2021 protocol—a Brazilian multicenter research protocol in which our institution participates—has been used. As a result, patients in this study followed two different protocols. The backbone strategy for both protocols is similar, though there are differences in the criteria used for risk classification. The GBTLI 2021 protocol reduces the intensity of induction therapy for patients classified as low and intermediate risk (Supplementary Frames S1, S2, and S3).

### 2.3 Sample collection

Bone marrow samples were collected via aspiration to confirm ALL diagnosis. Immediately after collection, smears were prepared for morphological evaluation. Bone marrow aspirate samples (or peripheral blood in cases of high white blood cell counts and patient severity) were sent to the Translational Research Laboratory for immunophenotyping by flow cytometry, cytogenetics (cell culture), and molecular biology analysis.

### 2.4 Minimal residual disease (MRD) assessment

MRD was evaluated by flow cytometry according to Euroflow Consortium guidelines (50). Cellular events were acquired using the FACS Canto II cytometer (BD), with data analysis performed using FACS Diva (BD) and Infinicyt (Cytognos, version 2.0) software. MRD values were measured at the mid-point and conclusion of the induction phase. For patients treated with the adapted ALLIC BFM 2009 protocol, MRD was assessed on days 15, 33, and 78. For patients following the GBTLI 2021 protocol, MRD quantification was conducted on days 19, 26 (for the low-risk subgroup), and 49.

### 2.5 Isolation of mononuclear cells and nucleic acid extraction

Mononuclear cells were isolated using a Ficoll gradient (GE Healthcare Life Sciences), washed in 1X PBS, and aliquoted into two aliquots. One tube was used for total RNA extraction via the Trizol® method (Invitrogen), and the other for DNA extraction. DNA was extracted using the Wizard Genomic DNA Purification Kit (Promega) following manufacturer instructions. Samples were quantified via spectrophotometry and stored at −20°C (for DNA) and −80°C (for RNA) until assays were performed.

### 2.6 Analysis of *FLT3* mutations

Screening for genetic variants in the *FLT3* tyrosine kinase domain (D835) was conducted on all patients with available samples (*n* = 155/156) via restriction fragment length polymorphism (RFLP) analysis (51). The PCR reaction included 1x PCR buffer, 200 nM each dNTP, 1.5 mM MgCl₂, 0.75 U Platinum™ Taq DNA Polymerase (Thermo Fisher Scientific), and 0.2 mM of each primer (FWD-CCGCCAGGAACGTGCTTG, REV-CAGCCTCACATTGCCCC). PCR conditions were as follows: 95°C for 3 min, 35 cycles of 95°C for 30 s, 56°C for 30 s, and 72°C for 1 min, followed by a final extension at 72°C for 5 min. PCR products were digested using the restriction enzyme EcoRV (NEB), and samples showing alterations by RFLP were subjected to Sanger sequencing.

Detection of genetic variants in the juxtamembrane domain was performed by fragment analysis (51) on the ABI3500 Genetic Analyzer (Applied Biosystems). PCR was performed under the same conditions described above, but with 27 cycles, using FWD-6-FAM-GCAATTTAGGTATGAAAGCCAGC and REV-CTTTCAGCATTTTGACGGCAACC primers. Fragment size was estimated using the GeneScan™ 500 LIZ size standard (Thermo Fisher Scientific).

For the classification of somatic variant pathogenicity in cancer (oncogenicity), we applied the Standard Operating Procedure developed in accordance with recommendations from the Clinical Genome Resource (ClinGen), the Cancer Genomics Consortium (CGC), and the Variant Interpretation for Cancer Consortium (VICC) (52).

### 2.7 Analysis of *FLT3* expression

*FLT3* expression was analyzed in patients admitted between July 2018 and December 2022 with available samples (*n* = 112/130) using real-time RT-PCR (RT-qPCR) on bone marrow samples collected at diagnosis. *FLT3* expression was also evaluated in in 10 samples collected at relapse. One microgram of RNA was used for cDNA synthesis, followed by PCR with 200 nM primers and 2x PCR MasterMix containing SybrGreen®. Relative quantification was performed using the 2-*ΔΔ*Cq method, with HPRT1 and B2M as reference genes. The calibrator was the median *Δ*Cq from all cases in the study, excluding relapses. Singleplex reactions were conducted on a QuantStudio 5 Real-Time PCR System (Thermo Fisher Scientific) with primers for *FLT3* (FWD-AGGGACAGTGTACGAAGCTG; REV-GTCGTGCTTAAAGACCCAGAG), HPRT1 (FWD-TGACACTGGCAAAACAATGCA; REV-GGTCCTTTTCACCAGCAAGCT), and B2M (FWD-TGCTGTCTCCATGTTTGATGTATCT; REV-TCTCTGCTCCCCACCTCTAAGT). Amplification conditions were: 2 min at 50°C, 10 min at 95°C for the holding stage, followed by 40 cycles of 95°C for 15 s and 60°C for 1 min. Melt curve analysis was performed to assess amplicon specificity.

### 2.8 Statistical analysis

Data were tested for normal distribution, by the D'Agostino and Pearson normality test and analyses of skewness and kurtosis, when applicable. Data were expressed as average ± SD (qPCR), mean ± SEM or median and ranges according to the distribution. Statistical analysis was performed using GraphPad Prism version 5.00 for Windows (GraphPad Software, San Diego California USA). The statistical approach adopted for each analysis is described in the figure legends. Non-parametric tests were used for data with non-normal distribution and probability values of p < 0.05 were accepted as indication of statistically significant difference.

### 2.9 Ethics

The study was approved by the local research ethics committee (protocol code 44796221.9.0000.0144. July 04, 2021). Informed consent was obtained from guardians, and assent forms were signed by patients over 5 years old.

## 3 Results

### 3.1 Characterization of the study population

**Figure 1:**
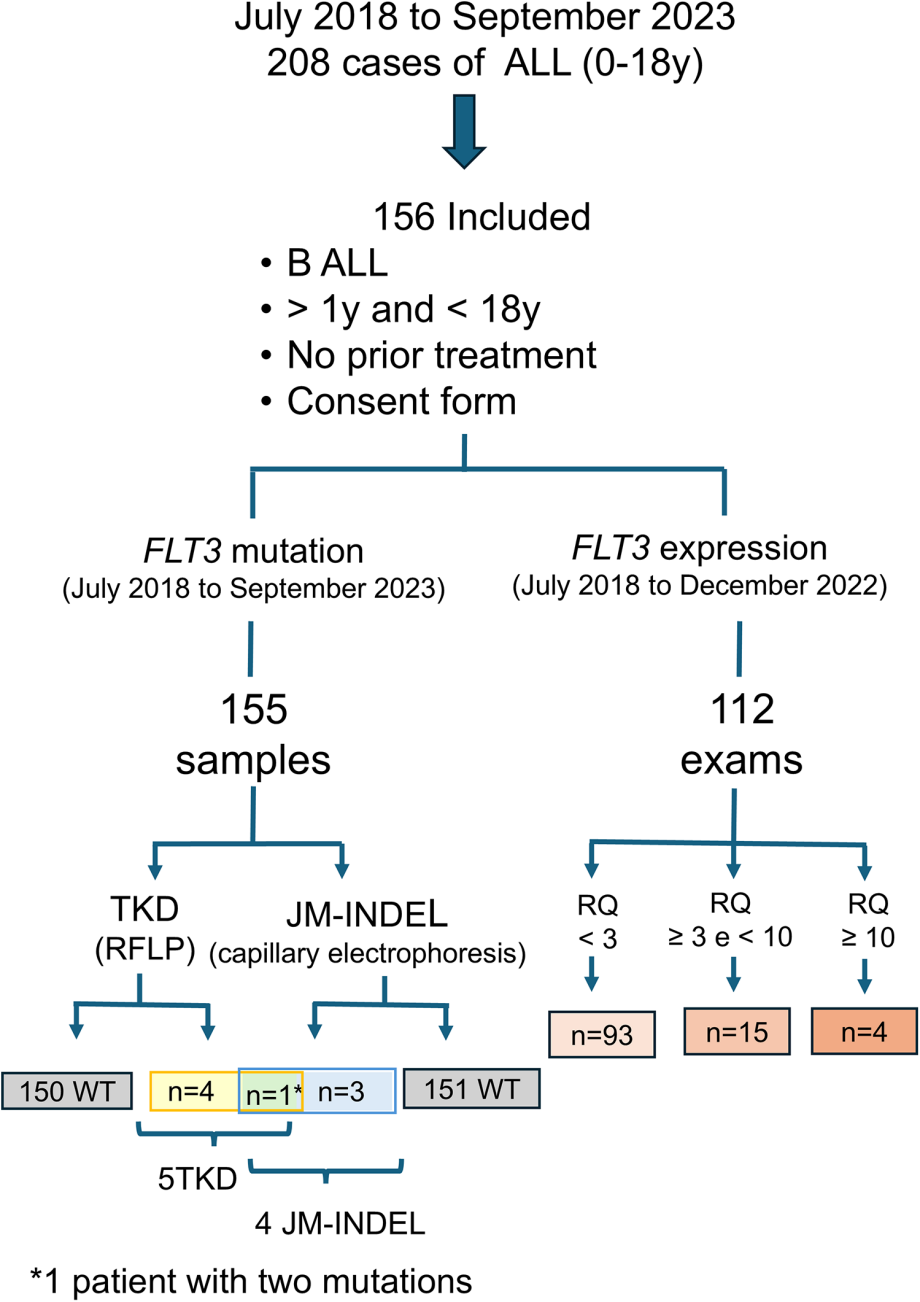
*FLT3* mutations and expression assessment in 156 children with B ALL diagnosis (RQ: relative quantification).

A total of 208 patients diagnosed with B-ALL were treated at our institution from July 2018–September 2023, with 156 meeting the inclusion criteria for this study (Figure 1). Patient characterization included age, sex, CNS status, treatment protocol applied, initial and post-induction risk classification, MRD values at mid-induction and post-induction, disease progression and current clinical status, cytogenetic alterations, recurrent rearrangements, presence of iAMP21, *IKZF1* deletions (whether associated with the *IKZF1* Plus subtype or not), *PAX5* alterations, *P2RY8::CRLF2* rearrangement, *FLT3* mutations, and *FLT3* expression levels.

The patients' ages ranged from 1–17 years, with a peak incidence between ages 2 and 5, and a median age of 4 years and 5 months. Thirty-four patients (21.8%) were over 10 years old at admission, and the sex ratio was 1.05 (80 males to 76 females). Regarding white blood cell counts at admission, 133 patients (85.2%) presented counts below 50,000 leukocytes/mm³, while 33 patients had counts above 50,000/mm³, including 12 with counts exceeding 100,000/mm³.

Most children (122) received treatment following the ALLIC BFM 2009 protocol. In terms of risk classification at the end of induction, 25 patients (20.4%) were classified as low risk, 74 (60.6%) as intermediate risk, and 23 (18.8%) as high risk. Among those treated under the GBTLI 2021 protocol, risk classifications were as follows: 16 (47%) intermediate risk, 12 (33%) high risk, 4 low risk, and 2 very high risk (Supplementary Table S1). Differences in risk classification criteria and treatment intensity between the two protocols led to a higher frequency of “high-risk” patients under the GBTLI protocol; however, this difference does not imply greater disease aggressiveness in this group.

**Figure 2:**
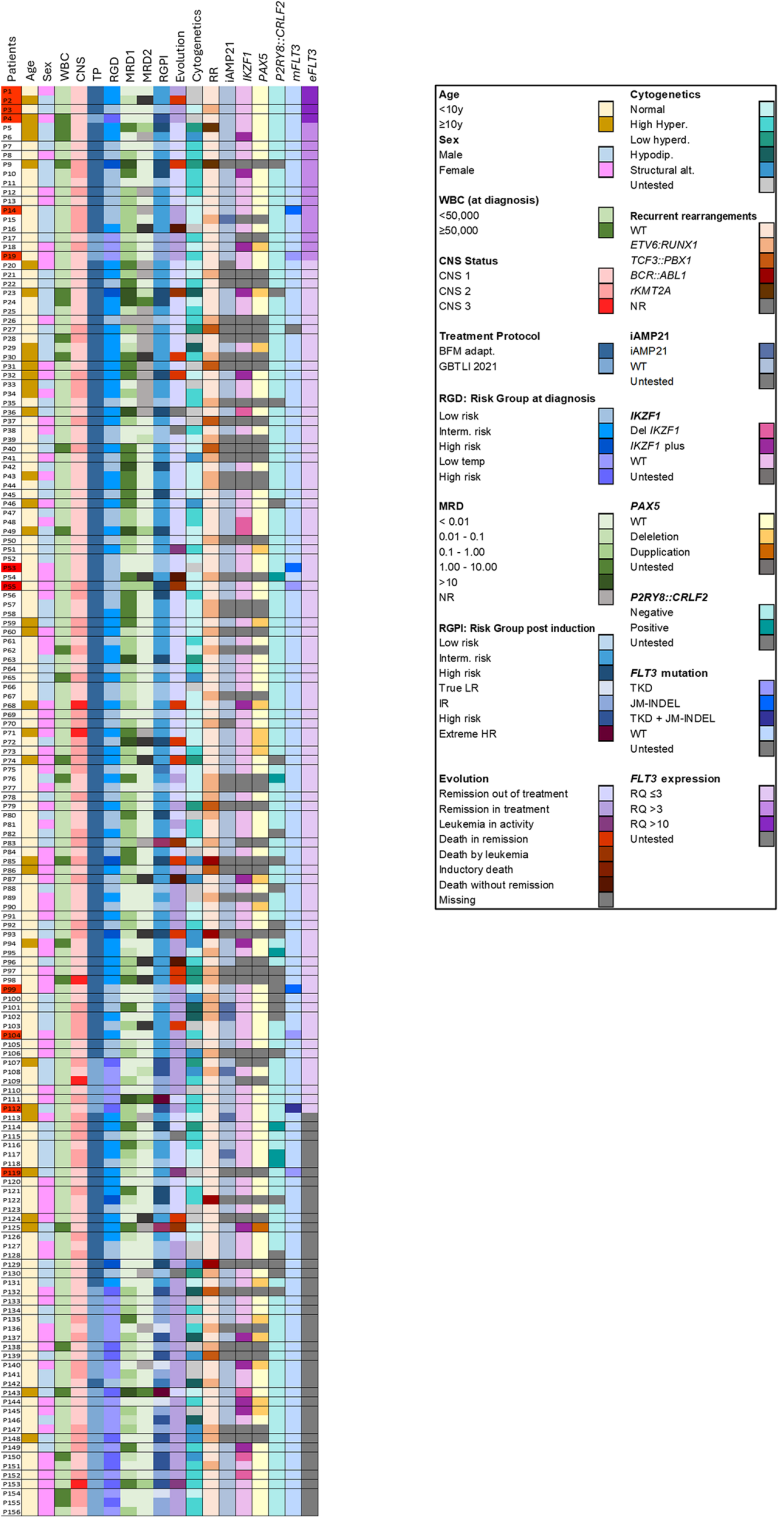
Clinical and molecular profile of the 156 patients included in the study. Interm. Risk: intermediary risk. Low temp: low risk temporary (GBTLI protocol). True LR: true low risk (GBTLI protocol). IR: intermediary risk. Extreme HR: extreme high risk. High hyperd: high hyplerdiploidy; Low hyperdip: Low hyperdiploidy. Hipodip: hypodiploidy. Strutural alt: structural alteration. m*FLT3*: *FLT3* mutation. *eFLT3*: *FLT3* expression. In red, in the left column, patients with *FLT3* overexpression (RQ > 10) or mutation are highlighted.

The 5-year overall survival (OS) and event-free survival (EFS) rates for this cohort were 87.5% and 78%, respectively, based on a minimum 5-year follow-up for 24 patients from the time of diagnosis. The clinical and laboratory data of the patients are presented in Figure 2.

### 3.2 *FLT3* mutations

**Figure 3:**
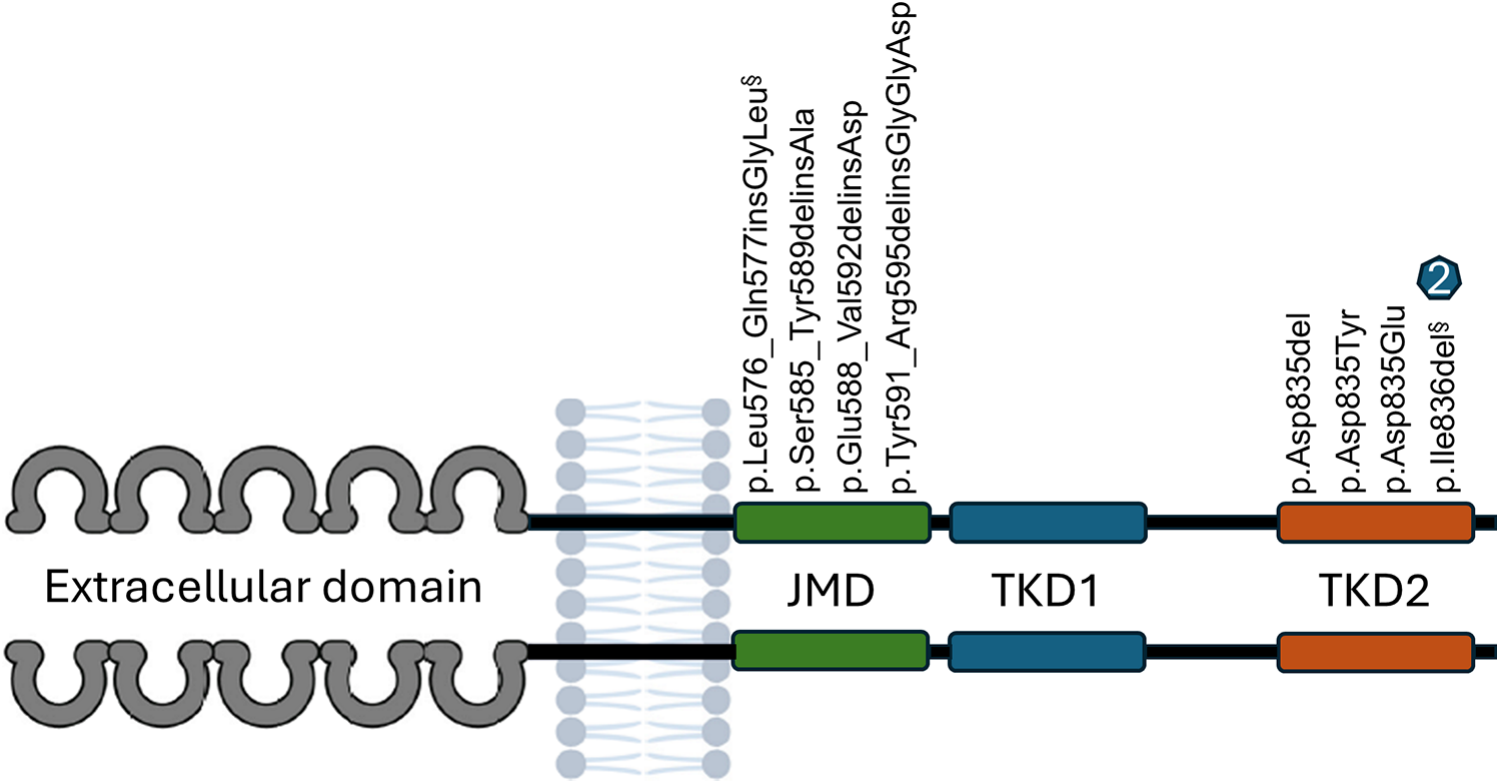
Description of eight distinct *FLT3* Gene Mutations in eight (8) patients with B-ALL (nine occurrences). *FLT3*-JM-INDEL mutations were identified in four patients. Five patients had mutations in the tyrosine kinase domain (*FLT3*-TKD). One patient exhibited two mutations: one in the juxtamembrane domain (§) and another in the tyrosine kinase domain (§).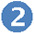 Two patients presented the p.Ile836del mutation, with one patient having this mutation exclusively and the other having it in conjunction with a *FLT3*-JM-INDEL (§).

*FLT3* mutation screening was conducted in 155 patients (Figure 1), revealing nine mutations in eight patients (5.1%). One type of mutation in the tyrosine kinase domain (p.Ile836del) was identified in two patients. Four patients (2.58%) presented with *FLT3*-TKD mutations, three patients (1.93%) had in-frame insertions and deletions in the juxtamembrane domain (*FLT3*-JM-INDEL), and one patient (0.64%) had mutations in both the tyrosine kinase domain (*FLT3*-TKD) and the juxtamembrane domain (*FLT3*-JM-INDEL) (Figure 3). No *FLT3*-ITD variants were found in our cohort.

The four mutations identified in the tyrosine kinase domain had been previously documented. Among these, three mutations have been associated with gain of function and oncogenic potential, while one mutation is likely to exhibit oncogenic potential due to a probable gain of function. None of the four mutations identified in the juxtamembrane region had been reported prior to this study. These alterations demonstrated moderate oncogenic potential attributed to a possible gain of function (Table 1).

**Table 1:** Description of *FLT3* mutations in patients with B-ALL.

Variant	Domain	Oncogenicity	Effect	Experimental Evidence	Reported in pediatric B-ALL
Leu576_Gln577insGlyLeu	JM	Moderate Oncogenic	Inframe change with probable	No evidence reported	First time reported in this work
		Support 4	Gain-of-Function		
Ser585_Tyr589delinsAla	JM	Moderate Oncogenic	Inframe change with probable	No evidence reported	First time reported in this work
		Support 5	Gain-of-Function		
Glu588_Val592delinsAsp	JM	Moderate Oncogenic	Inframe change with probable	No evidence reported	First time reported in this work
		Support 5	Gain-of-Function		
Tyr591_Arg595delinsGlyGlyAsp	JM	Moderate Oncogenic	Inframe change with probable	No evidence reported	First time reported in this work
		Support 4	Gain-of-Function		
Asp835del	TKD2	Oncogenic	Gain-of-Function	Clark, et al., Blood (64) (PMID:15256420)	Zhao, et al., J. Mol. Sci (20) (PMID:39273530).
Asp835Tyr	TKD2	Oncogenic	Gain-of-Function	Yamamoto, et al., Blood (13). (PMID:11290608)	Spinella et al., BMC Cancer (65). (PMID:26345285).
					Zhang et al., Nat Genet (2). (PMID:27776115).
				Clark, et al., Blood (64) (PMID:15256420)	Roberts et al., Engl J Med (53). (PMID:25207766).
					Zhang, et al., Cancer Gene Ther (21). (PMID:31285539)
					Gutierrez-Camino, *et al*., Br. J. Cancer (36). PMID:38049555)
				Bailey, et al., PNAS (66) (PMID:24255108)	Zhao, et al., J. Mol. Sci (20). (PMID:39273530).
Asp835Glu	TKD2	Oncogenic	Gain-of-Function	Yamamoto, et al., Blood (13). (PMID:11290608).	Zhang, et al., Cancer Gene Ther (2) (PMID:31285539).
					Gutierrez-Camino, et al., Br. J. Cancer (36) (PMID:38049555).
				Clark, et al., Blood (64) (PMID:15256420).	Zhao, et al., J. Mol. Sci (20) (PMID:39273530).
Ile836del	TKD2	Likely Oncogenic	Likely Gain-of-Function	Grundler, et al., Blood (67) (PMDI:12663439).	Ma, et al., Nature 2018 (PMID:29489755).
					Zhang, et al., Cancer Gene Ther (21) (PMID:31285539).
				Clark, et al., Blood (64) (PMID:15256420).	Newman, et al., Cancer Discov 2021 (PMID:34301788).
					Zhao, et al., J. Mol. Sci (20) (PMID:39273530).

JM, juxtamembrane domain; TKD, tyrosine kinase domain. Platforms used for search: COSMID, HGMD, cBioPortal, OncoKB, NCBI, LOVD.

No association was found between *FLT3* mutations and age, gender, white blood cell (WBC) count at diagnosis, or minimal residual disease (MRD) values measured at the mid-point and at the end of induction therapy. Table 2 presents the clinical features and detailed descriptions of the *FLT3* mutations.

**Table 2:** Characteristics of patients with *FLT3* mutations.

No	Age (years)	Gender	CNS	WBC	Protocol	MRD D15 or D19	MRD D33	MRD D78 or D49	Risk Group	Event	Current Status	Association	Mutation	Expression (RQ)	Description
P14	2	M	CNS 2	<5,000	Adapted BFM	>0.1–<1.0	0	NR	IR	0	RIT	–	JM-INDEL	3.077	c.1771_1785delinsGGTGGGGAC
															p. Tyr591_Arg595delinsGlyGlyAsp
P19	5	F	CNS 1	<5,000	GBTLI 2021	>0.1–<1.0	NR	0	IR	0	RIT	HHD	TKD	4.082	c.2505T > G;
															p. Asp835Glu
P53	2	F	CNS 1	10,000–50,000	Adapted BFM	0	0	0	LR	0	ROT	–	JM-INDEL	1.491	c.1764_1775del
															p. Glu588_Val592delinsAsp
P55	3	F	CNS 2	5,000–10,000	Adapted BFM	>0.1–<1.0	>0.01–<0.1	>0.1–<1.0	HR	Rel	DL	–	TKD	2.533	c.2508_2510del
															p. Ile836del
P99	1	F	CNS 1	5,000–10,000	Adapted BFM	<0.01	0	0	LR	0	RIT	–	JM-INDEL	2.847	c.1753_1766delinsGC
															p. Ser585_Tyr589delinsAla
P104	9	F	CNS 2	<5,000	Adapted BFM	0	0	0	IR	0	RIT	HHD	TKD	0.981	c.2503_2505del
															p. Asp835del
P112	17	M	CNS 1	10,000–50,000	GBTLI 2021	>0.1–<1.0	NR	0	HR	0	RIT	HHD	TKD/	2.276	c.2508_2510del
															p.Ile836del
													JM-INDEL		
															c.1727_1728insGGGGCT
															p. Leu576_Gln577insGlyLeu
P119	11	M	CNS 1	<5,000	Adapted BFM	>0.01–<0.1	0	0	IR	Rel	2nd relapse	Untested	TKD	NR	c.2503G > T
															p.Asp835Tyr

CNS, central nervous system; WBC, white blood cells; MRD, minimal residual disease; IR, intermediate risk; LR, Low risk; HR, High risk; Rel., relapse; ROT, remission out of treatment; DL, death for leukemia; RIT, remission in treatment; HHD, High hyperdiploidy.

*FLT3* mutations were associated with high hyperdiploidy in 3 out of 8 patients (37.5%) (patients P19, P104, P112,). All three cases had TKD-type mutations, and one patient presented with a JM-INDEL associated with a TKD mutation (P112). No *FLT3* mutations were identified in any patients with *ETV6::RUNX1* (*n* = 38), *BCR::ABL1* (*n* = 4), *TCF3::PBX1* (*n* = 9), or r-*KMT2A* (*n* = 2). Additionally, none of the patients with *P2RY8::CRLF2* (*n* = 7), *IKZF1* deletions (*n* = 21), including *IKZF1*plus (*n* = 11), *PAX5* alterations (*n* = 21), or *ERG* deletions (*n* = 9) presented with *FLT3* mutations (Table 2, Figure 2).

**Figure 4:**
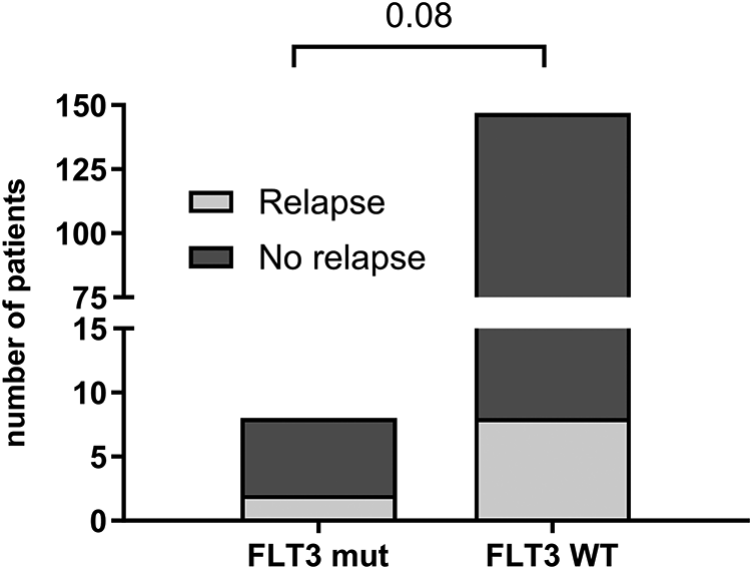
Relapse occurrence in patients with *FLT3* gene mutation (*n* = 8) and wild type—WT (*n* = 147). Statistically analyzed by Fisher's exact test.

Among patients with *FLT3* mutations, a relapse rate of 25% (2/8) was observed, with both cases occurring in patients with the *FLT3*-TKD mutation and no associated high hyperdiploidy. Although this difference was not statistically significant compared to *FLT3* wild-type (WT) patients (*p* = 0.08), it is noteworthy given the rarity of the mutation. The relapse rate among *FLT3* WT patients was 5.4% (8/147) (Figure 4).

Follow-up of the two relapsed patients with *FLT3*-TKD mutations revealed that one experienced a very early relapse, failed to achieve remission, and died three months after the relapse diagnosis (*FLT3* mutation screening was not conducted on the relapse sample). The other patient achieved remission but experienced a second relapse with an orbital lesion. Treatment included chemotherapy and orbital radiotherapy (20 Gy). The mutation in *FLT3* identified in the initial diagnosis sample was also detected in the bone marrow sample collected at the time of relapse. This patient is currently alive, with a follow-up period of 55 months.

**Figure 5:**
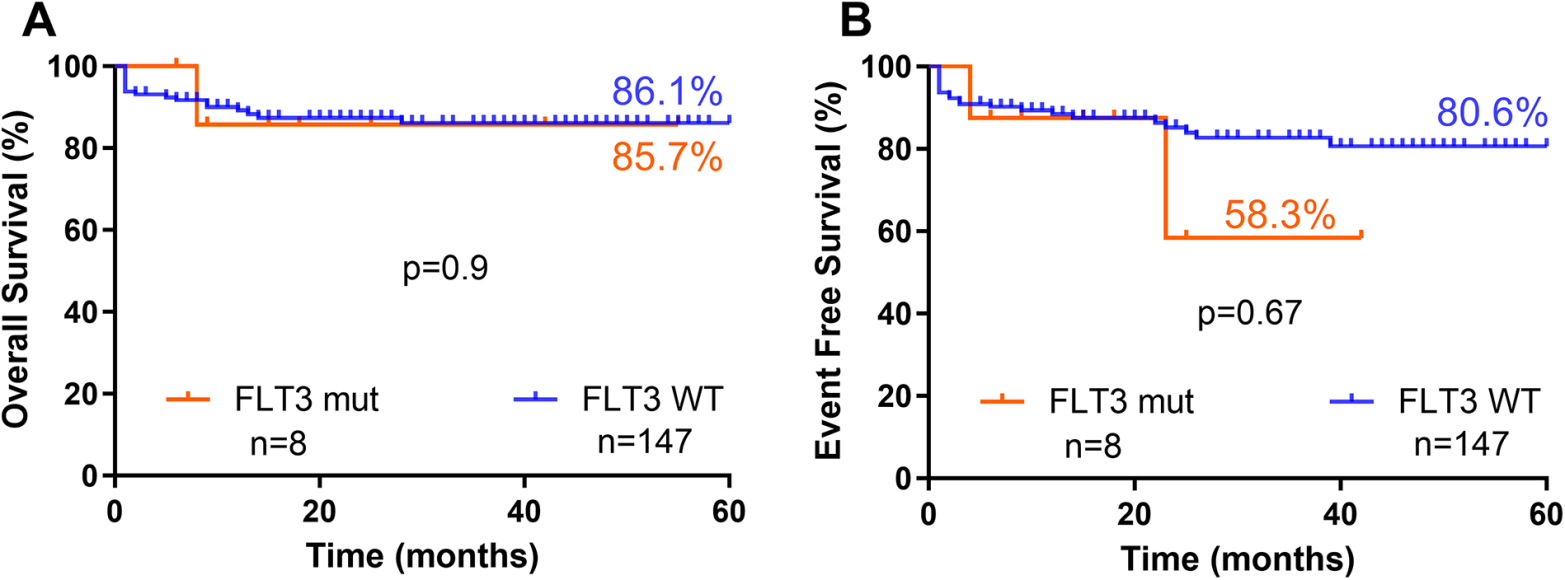
Survival curves for B-ALL pediatric patients according to status of *FLT3* mutation **(A)** (global survival) and **(B)** (event free survival). Statistically analyzed by Log-rank (Mantel-Cox) test.

There was no difference in survival rates when comparing patients with *FLT3* mutations to those with *FLT3* WT (Figure 5).

### 3.3 *FLT3* expression

*FLT3* expression was evaluated in RNA samples of 112 patients at diagnosis. Additionally, *FLT3* expression was assessed in 10 samples collected at relapse.

**Figure 6:**
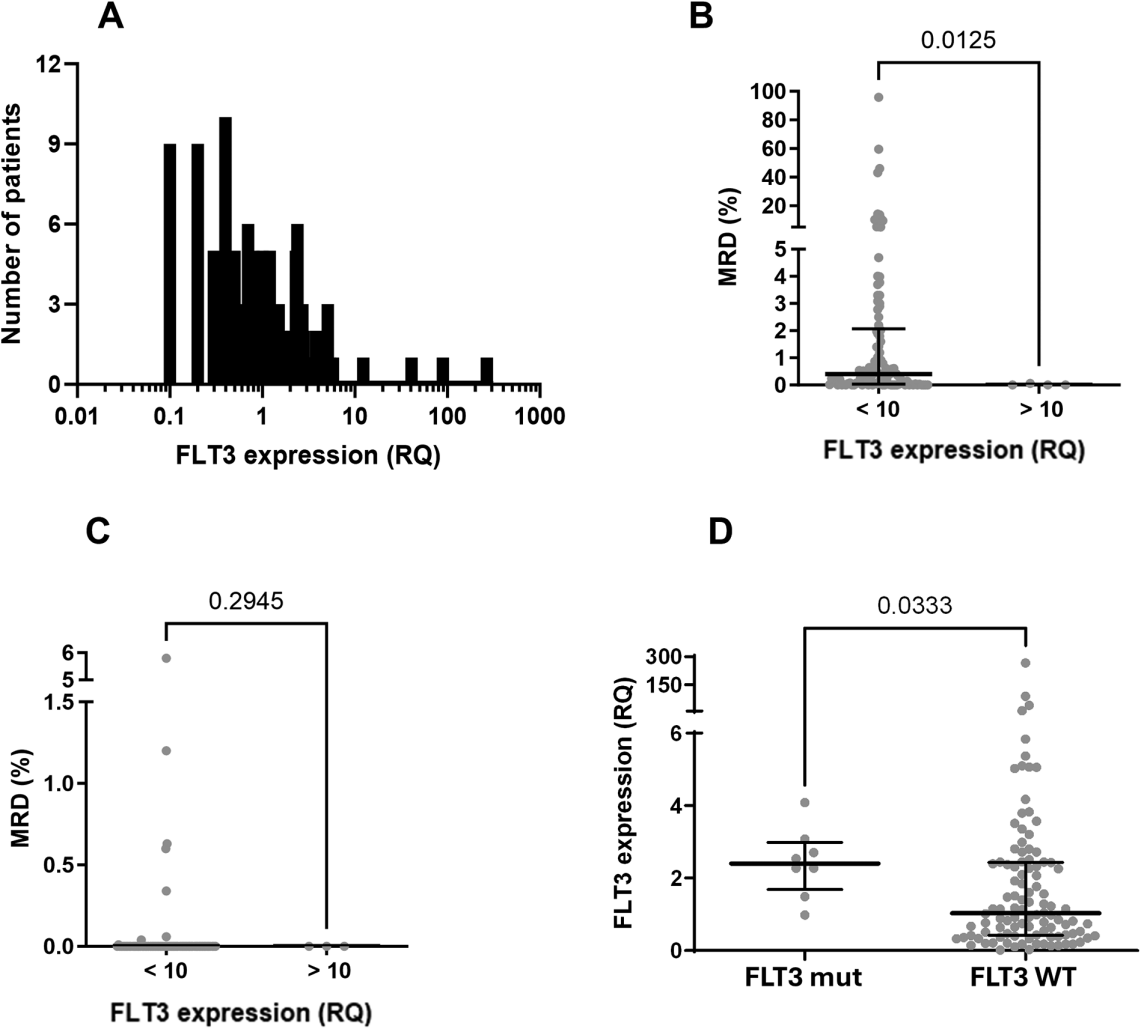
*FLT3* expression analysis in samples of children with B-cell ALL. **(A)** Frequence distribution of *FLT3* expression (relative quantification—RQ) in the studied population (attention to the log scale). Data did not pass in Shapiro-Wilk normality test and showed skewness of +8.69. **(B)** Evaluation of MRD at mid-induction (D15 for patients treated with the adapted BFM ALLIC 2009 protocol and D19 for patients treated with the Brazilian GBTLI 2021 protocol) among patients overexpressing *FLT3* (>10) and all the others (<10). Statistical analyses were performed with Mann Whitney test. **(C)** Evaluation of MRD at the end of induction (D78 for patients treated with the BFL ALLIC 2009 and D49 for the group treated with the GBTLI protocol) among patients overexpressing *FLT3* (>10) and all the others (<10). Statistical analyses were performed with Mann Whitney test. **(D)** *FLT3* expression between patients with (*FLT3* mut) and without (*FLT3* WT) mutations. Statistical analyses were performed with Mann Whitney test.

The distribution of relative *FLT3* expression in the samples showed a non-Gaussian pattern, with a strong rightward skew (Figure 6A), indicating high expression levels without correspondingly low levels, as would be expected for independent variables with a normal distribution. This pattern is typical of genes with biological significance in carcinogenesis, where elevated expression levels confer an advantage, and lower levels are incompatible with disease maintenance (54, 55).

In 93 patients, the expression ratio (RQ) ranged from 0.017–3. Fifteen patients had expression ratios between 3 and 6.13, and in four patients, the expression ratio exceeded 10, which we classified as “hyperexpression.” These elevated RQ values were observed exclusively in four wild-type (WT) *FLT3* patients. We were unable to identify factors associated with *FLT3* hyperexpression in these four patients with RQ > 10 (see Table 3), but we observed that these individuals demonstrated a rapid response to treatment, as indicated by MRD values assessed during mid-induction (D15 or D19, depending on the protocol) (Figure 6B). Conversely, there was no statistically significant difference in MRD values at the end of induction between patients with hyperexpression and those without (Figure 6C).

**Table 3:** Characteristics of patients with *FLT3* overexpression.

	Age (years)	Gender	CNS	WBC	Protocol	MRD D15/D19	MRD D33	MRD D78/D49	Risk Group	Event	Current Status	Association	Mutation	Expression (RQ)
P1	4	F	CNS1	10,000–50,000	Adapted BFM	<0.01	0	0	IR	0	RIT	–	WT	266.6
P2	17	M	CNS1	<5,000	Adapted BFM	>0.01–<0.1	0	NR	IR	Death in remission	DR	DEL CDKN2A 2B	WT	89.3
P3	3	M	CNS1	<5,000	Adapted BFM	<0.01	0	0	LR	0	RIT	*ETV6::RUNX1*	WT	40.8
P4	13	F	CNS1	>1,00,000	GBTLI 2021	<0.01	NR	0	HR	0	RIT	–	WT	12.3

CNS, central nervous system; WBC, white blood cells; MRD, minimal residual disease; IR, intermediate risk; LR, Low risk; HR, High risk; DR, death in remission; RIT, remission in treatment.

Although none of the patients with *FLT3* mutations exhibited hyperexpression of the gene (RQ > 10), *FLT3* expression values were higher among patients with *FLT3* mutations compared to wild-type patients (median 2.53 vs. 1.03, *p* = 0.03) (Figure 6D).

No significant differences in *FLT3* expression values were observed among patients across different risk groups (data not shown).

**Figure 7:**
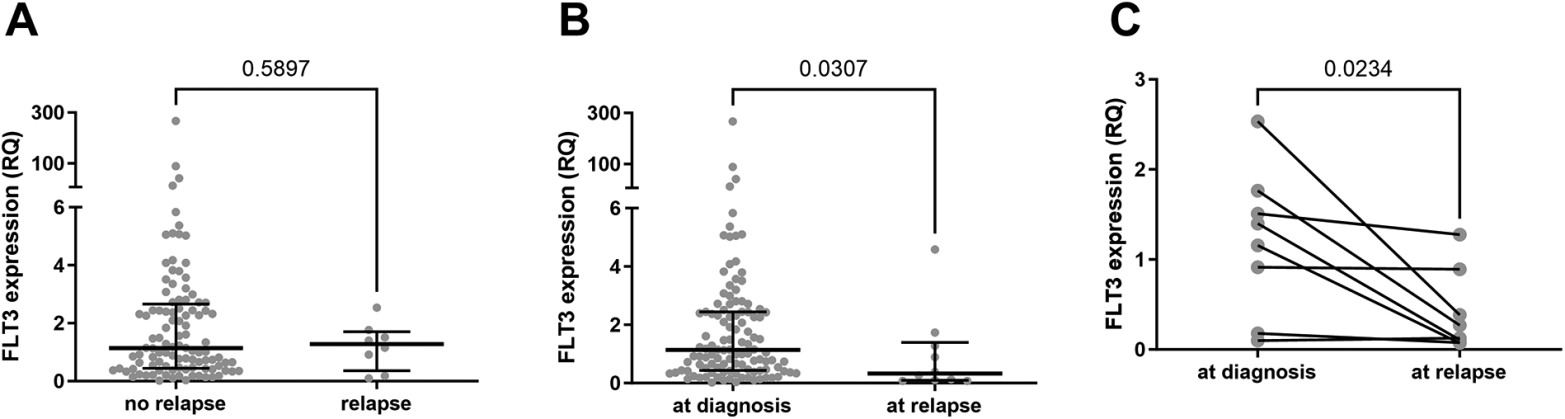
*FLT3* expression levels analysis in relapsed patients vs. patients in remission. **(A)** Comparison between *FLT3* expression values of samples from 103 patients initially diagnosed with B-cell ALL who did not experience relapse until last data assessment and *FLT3* expression values in BM samples collected at the initial diagnosis from 8 patients who relapsed. Statistically analyzed by Mann Whitney test. **(B)** Comparison between *FLT3* expression values of samples from 112 patients initially diagnosed with B-cell ALL and *FLT3* expression values of BM samples collected at the time of relapse from 10 patients. Statistically analyzed by Mann Whitney test. **(C)** *FLT3* expression levels of 8 samples from patients at the initial diagnosis and at the time of relapse. The patient with the highest RQ value (2.5) at diagnosis is the only one in the group with *FLT3* mutation. Statistically analyzed by Wilcoxon matched-pairs signed rank test.

**Figure 8:**
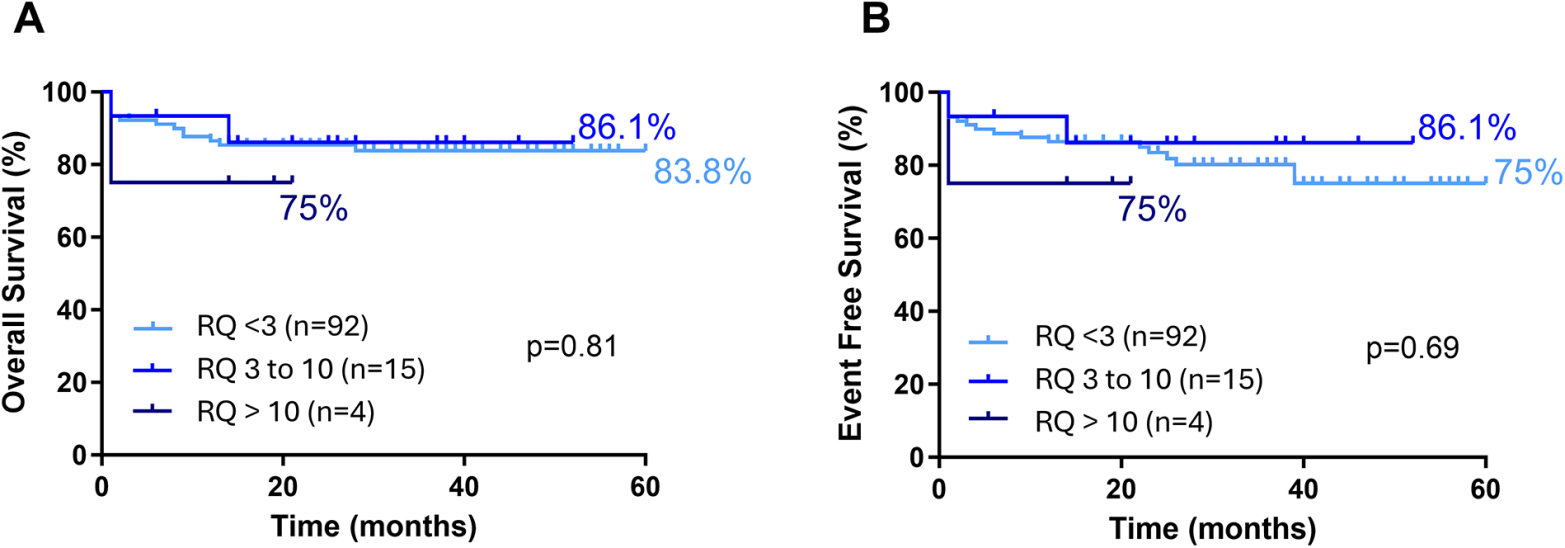
Survival curves for B ALL pediatric patients according to *FLT3* expression status. **(A)** (Overal Survival) and **(B)** (Event free survival). Statistically analyzed by Log-rank (Mantel-Cox) test.

A noteworthy finding is that *FLT3* expression levels were significantly lower in relapse cases. Patients who relapsed had relative expression levels (at initial diagnosis) comparable to the group median (Figure 7A). However, these values decreased markedly after relapse (Figure 7B). This difference is even more pronounced when comparing paired expression values at diagnosis and at relapse for each patient (Figure 7C). Despite this, *FLT3* expression levels had no significant impact on survival rates (EFS and OS) (Figure 8, Supplementary Table S2).

## 4 Discussion

Limited studies have investigated the implications of the *FLT3* gene in B-cell Acute Lymphoblastic Leukemia (B-ALL), likely due to the infrequent occurrence of *FLT3* alterations. Nonetheless, in recent years, *FLT3* has emerged as a significant marker for enhancing the biological characterization of patients with ALL, particularly within specific subtypes such as Ph-like and r-*KMT2A* ALL (31, 32, 44, 53, 56). Furthermore, several researchers have emphasized the need for additional studies to elucidate the role of *FLT3* in B-ALL patients (20, 31, 33). Although this study is based on data from a single institution, it complements and builds upon previous research. Moreover, it is notable for evaluating a population with a distinct genetic composition, characterized by a mixture of various ethnic backgrounds (indigenous, African, Caucasian).

### 4.1 *FLT3* mutations

In our study, we found *FLT3* mutations in 5.1% of the samples (8/155), with 3.2% (5/155) of mutations occurring in the TKD domain (in one case, associated with JM-INDEL). Unlike acute myeloid leukemia (AML), the variants found in the juxtamembrane domain were indels rather than internal tandem duplications (ITDs).

These findings are consistent with those reported in previous studies regarding both the frequency of mutations and the different spectrum of *FLT3* mutations in B-cell acute lymphoblastic leukemia (B-ALL) compared to AML (20, 21). Taketani *et al*. (2004) found *FLT3*-TKD mutations in 6 (5.4%) of 112 children with ALL older than 1 year and in 8 (16.0%) of 50 infants with ALL, but no *FLT3*-ITD mutations were detected (32). Zhang *et al*. (2020) reported a predominance of JM-INDELs in B-ALLs (21). In Saudi Arabia, 4.7% of children with B-ALL had *FLT3* mutations (2.4% ITD) (33), and in Brazil, Barbosa *et al*. found *FLT3* mutations in 6.7% of 134 B-ALL patients (34). Additionally, this frequency was reported to be 5.5% among Canadian children and adolescents with ALL, including 1.1% *FLT3*-ITD and 4.3% *FLT3*-TKD point mutations, with an observed association between *FLT3* mutations and hyperdiploidy (36).

We recognize that broader horizontal coverage and the ability to simultaneously detect multiple genetic alterations [single nucleotide variants [SNVs], copy number variations [CNVs], fusions, and indels] using highly sensitive techniques like next-generation sequencing (NGS) allow for the identification of *FLT3* mutations at higher frequencies than restriction fragment length polymorphism (RFLP) and fragment analysis (20, 44). While NGS provides comprehensive genomic insights, it is time-consuming and may produce false negatives due to amplification issues or software limitations in detecting *FLT3* internal tandem duplications (*FLT3*-ITD). In contrast, PCR fragment analysis and PCR-RFLP targeting *FLT3*-TKD (D835/I836) mutations are robust, cost-effective methods that deliver faster results, making them a viable and efficient alternative to NGS (57, 58).

In a recently published study, Zhao *et al*. described a higher frequency of *FLT3* mutations in ALL patients when evaluated by NGS (6.3% ITD and 18.8% TKD) and identified new non-canonical genetic variants, such as point mutations outside the TKD and insertion/deletion variants causing in-frame amino acid alterations. In the same study, a higher proportion of patients with negative minimal residual disease (MRD) at mid-induction (D19) was observed among patients with *FLT3* mutations compared to wild-type patients (20). In our sample, we did not identify differences in MRD levels between patients with or without *FLT3* mutations.

Regarding initial presentation, unlike what is well established in patients with AML, where *FLT3* mutations are associated with elevated leukocyte counts at diagnosis (22, 23, 59), none of the patients with *FLT3* mutations in our study presented with hyperleukocytosis. Furthermore, no patients with *FLT3* mutations had central nervous system (CNS) involvement at diagnosis.

In our sample, *FLT3* mutations were not detected in patients with recurrent genetic alterations commonly associated with B-ALL, including *ETV6::RUNX1*, *TCF3::PBX1*, *BCR::ABL1*, r-*KMT2A*, *P2RY8::CRLF2* rearrangements, *PAX5* alterations, or deletions involving *IKZF1*. This finding supports the hypothesis that, contrary to the prevailing concept of kinase alterations being secondary (31, 60), *FLT3* mutations may function as leukemogenic drivers in a small subset of B-ALL (5, 61). Further studies utilizing bone marrow samples collected during treatment monitoring or at relapse could clarify the role of *FLT3* mutations in disease progression. Preliminary studies suggest that *FLT3* mutations negatively impact prognosis in infants with r-*KMT2A* B-ALL (31, 49). However, in patients with hyperdiploidy, the presence of *FLT3* mutations did not affect the natural course of the disease in this subgroup, which generally has a favorable outcome (31–33). In our study, among the five patients with TKD mutations, three who also had hyperdiploidy achieved remission, while the other two without hyperdiploidy experienced relapse. In light of these results and based on evidence of an association between hyperdiploidy and higher levels of *FLT3* expression, as well as previous studies linking elevated *FLT3* expression with certain subtypes of B-ALL (r-*KMT2A*, Ph-like), we decided to complement our research by evaluating *FLT3* expression levels.

### 4.2 *FLT3* expression

Higher *FLT3* expression levels are described and considered recurrent alterations in acute leukemias. The expression levels of this gene are higher in cells from patients with acute leukemias compared to normal bone marrow samples and other types of neoplasms (37–43). In the context of B-ALL, subgroups of patients with r-*KMT2A*, high hyperdiploidy, and r-*ZNF384* subtypes exhibit higher *FLT3* expression levels (5, 36, 37, 40, 42). In our study, which was limited to patients older than one year, only two patients had r-*KMT2A*, both with expression values above the 75th percentile of our cohort.

Although the association between hyperdiploidy and higher levels of *FLT3* expression has been documented in some studies (44, 61, 62), we did not find differences in *FLT3* expression in this group. Similarly, there was no correlation between *FLT3* expression and indicators of aggressiveness in B-ALL, such as white blood cell count at diagnosis or CNS involvement. This lack of association may be specific to our study population; however, expanding the sample size is necessary to confirm these findings.

Additionally, we were unable to identify any common characteristics among the four patients with extremely high expression levels (RQ > 10). Yang and colleagues demonstrated that epigenetic alterations with enhancer hijacking secondary to the deletion of the *PAN3* gene (13q12.2) explain elevated *FLT3* expression in B-ALL patients, particularly among those with hyperdiploidy or those who experienced relapse. Although none of the four patients with RQ > 10 had associated hyperdiploidy or experienced disease relapse, this could be a possible mechanism to explore in these cases (42).

The implications of *FLT3* expression levels on prognosis remain uncertain and controversial. Among patients with r-*KMT2A* B-ALL, high *FLT3* expression levels have been associated with poorer outcomes (38, 40, 49). However, in 2017, Fedders and colleagues found an opposite association (63). In our study, we were unable to identify any influence on survival rates in patients with B-ALL and *FLT3* overexpression detected in bone marrow samples at diagnosis. The majority of authors also did not find an association between *FLT3* expression levels at diagnosis and survival or relapse rates (20, 36). On the other hand, Garza Veloz reported that high *FLT3* expression levels at the end of induction were associated with higher relapse and mortality rates (40).

We identified evidence that *FLT3* may be a marker influencing the biological behavior of B-ALL, reinforcing the need for more in-depth studies to better elucidate *FLT3*'s relationship with mechanisms involved in the genesis, survival, or resistance of leukemic cells to chemotherapeutic effects: 1. the frequency distribution of relative *FLT3* expression (Figure 6); 2. the low MRD values observed during mid-induction in patients with high *FLT3* expression (Figure 6); and 3. significantly lower *FLT3* expression levels in samples from relapsed patients (Figure 7).

Given these results, the detailed biological effects and prognostic impact of *FLT3* expression levels should be further investigated, particularly in the context of the potential use of FLT3 inhibitors. Additionally, it is important to not only focus on transcript expression levels but also evaluate the true functional impact of *FLT3* by assessing receptor saturation or activation.

### 4.3 Limitations

The small sample size, particularly given the low frequency of *FLT3* mutations, along with the limited follow-up time for the patients, constrains the interpretation of our findings and underscores the necessity for continued research in this area. We acknowledge the inherent limitations of Restriction Fragment Length Polymorphism (RFLP) and fragment analysis in detecting low-frequency *FLT3* mutations, especially when compared to the enhanced sensitivity of Next-Generation Sequencing (NGS).

Nevertheless, considering the potential for direct and short-term benefits for selected patients, we believe it is crucial to disseminate our findings, even with the limited follow-up period. In light of these limitations, we assert that our findings contribute meaningfully to the understanding of *FLT3* mutation prevalence and advocate for further research and investment in comprehensive molecular diagnostics in similar contexts.

### 4.4 Perspectives

Preclinical studies and case reports provide evidence supporting the use of FLT3 inhibitors in specific subgroups of B-ALL patients (3, 45, 46, 56). However, although the use of FLT3 inhibitors has been suggested as a therapeutic option for B-ALL patients for nearly two decades, studies exploring this approach remain scarce (46, 56).

We propose that in selected cases, alongside MRD assessment, evaluating *FLT3* status (mutation or expression) may serve as an additional tool in guiding treatment strategies. For instance, in patients with persistent MRD following induction or in relapse cases with *FLT3* mutations or increased expression, FLT3 inhibitors could be considered as salvage treatment. Another option is to use *FLT3* inhibitors in combination with conventional chemotherapy to reduce the required doses in patients with a favorable prognosis. FLT3 inhibitors may also be beneficial for patients with *FLT3* mutation or overexpression where high-dose chemotherapy is contraindicated, such as in cases of infection or toxicity.

## 5 Conclusion

In conclusion, although we did not find consistent data regarding the impact of *FLT3* mutations or elevated *FLT3* expression levels on patient response (measured by MRD values after induction) or relapse rates, our findings suggest that *FLT3* alterations — whether genetic or expression-related —exert a biological influence on the behavior of leukemic cells and may complement traditional tools used to enhance B-ALL characterization. Moreover, these alterations may help guide therapeutic strategies for selected B-ALL patients by utilizing FLT3 inhibitors.

Currently, there is no evidence supporting the inclusion of FLT3 inhibitors as a first-line therapeutic approach for B-ALL patients with *FLT3* alterations. However, we believe that in cases of persistent MRD positivity following induction therapy or in instances of relapse, the use of FLT3 inhibitors should be considered as salvage therapy for patients with *FLT3* mutations or elevated *FLT3* expression. An additional, somewhat more ambitious possibility worth exploring is the combination of FLT3 inhibitors with conventional treatment to reduce chemotherapy doses.

This hypothesis requires validation in larger cohorts and through studies utilizing samples collected at different stages of leukemia treatment. Despite these limitations, our study highlights a relatively unexplored aspect of B-ALL with promising translational potential, paving the way for more personalized treatment approaches.

## Data Availability

The authors acknowledge that the data presented in this study must be deposited and made publicly available in an acceptable repository, prior to publication. Frontiers cannot accept a manuscript that does not adhere to our open data policies.
