# Supplementary material for: Prevalence of FLT3 gene mutation and its expression in Brazilian pediatric B-ALL patients: clinical implications
Source: Front Pediatr. 2024 Dec 6;12:1505060. doi: 10.3389/fped.2024.1505060 (PMC11658997; doi:10.3389/fped.2024.1505060)
Supplement: Supplementary file 1 [file Datasheet1.pdf]

Supplementary Material

1 Supplementary Figures and Tables

1.1 Supplementary Figures

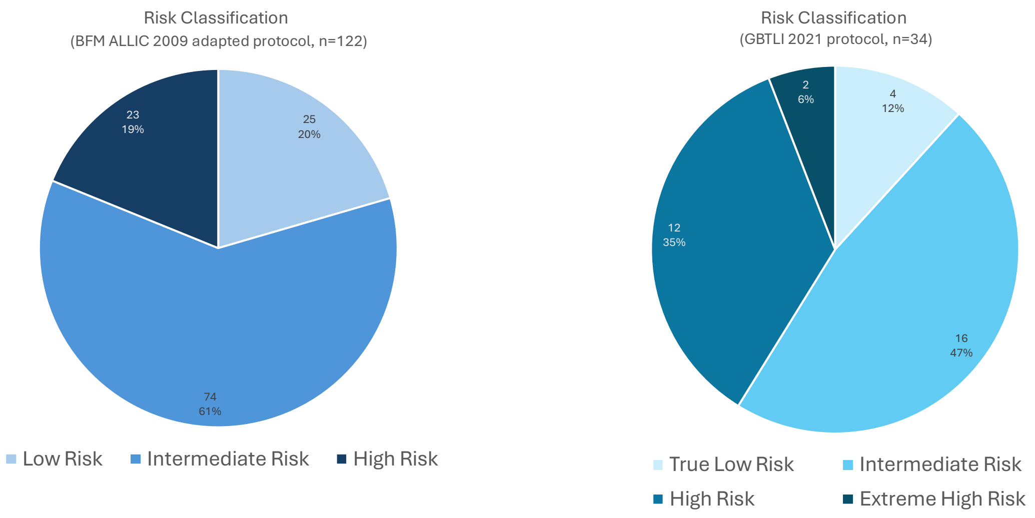

Supplementary Figure 1. Risk classification of B-ALL patients. A) Patients treated according BFM ALLIC 2009 adapted protocol (n=122). B) Patients treated according GBTLI 2021 protocol (n=34)

1.2 Supplementary Frames

Supplementary Frame 1. Induction Treatment of adapted ALLIC BFM 2009 and GBLT2021 protocols

**Treatment Protocol**	**BFM adapted**			**GBTLI 2021**		
**Risk Classification**	Low Risk	Intermediate Risk (IR)	High Risk (HR)	Provisional Low Risk (PLR)	Intermediate Risk (IR)	High Risk (HR)
**Risk Criteria at Diagnosis**	Age > 1yo and <6yo and<br>WBC<20000/mm³<br>and<br>No High Risk Crietria	No low risk and<br>No high-risk criteria	*BCR::ABL1*<br>*KMT2A::AF4*	Age ≥ 1 and < 10<br>WBC < 50000/mm³<br>CNS1 or CNS2<br>Absence of testicular infiltration	Previous use of corticosteroid<br>PLR and MRD D19 > 0.01	Age ≥ 10 years<br>WBC ≥ 50000<br>CNS3<br>Testicular infiltration<br>(*TCF3::PBX1*); (*KMT2A::AFF1*); Ph-like;<br>(*TCF3::HLF*); hypodiploidy < 45 chromosomes (DNA index < 1)
**Induction Chemotherapy**	Phase 1<br>VCR 1.5mg/m² X 4<br>Daunorrubicin 25mg/m² X 2<br>PEG asparaginase 2500UI/m² X 1<br>Prednisone 40mg/m² D1 a D28<br>MADIT X 3 (5X if CNS2)<br><br>Phase 2<br>Cyclophosphamide 1000mg/m² X 2<br>Cytarabine 75mg/m² X 4 days X 4 weeks<br>6MP 60mg/m²/day 28 days<br>MADIT 2X	Phase 1<br>VCR 1.5mg/m² X 4<br>Daunorrubicin 25mg/m² X 4<br>PEG asparaginase 2500UI/m² X 2<br>Prednisone 40mg/m² D1 a D28<br>MADIT X 3 (5X if CNS2)<br><br>Phase 2<br>Cyclophosphamide 1000mg/m² X 2<br>Cytarabine 75mg/m² X 4 days X 4 weeks<br>6MP 60mg/m²/day 28 days<br>MADIT 2X	Phase 1<br>VCR 1.5mg/m² X 4<br>Daunorrubicin 25mg/m² X 4<br>PEG asparaginase 2500UI/m² X 2<br>Prednisone 40mg/m² D1 a D28<br>MADIT X 3 (5X if CNS2)<br><br>Phase 2<br>Cyclophosphamide 1000mg/m² X 2<br>Cytarabine 75mg/m² X 4 days X 4 weeks<br>6MP 60mg/m²/day 28 days<br>MADIT 2X	Phase 1<br>VCR 1.5mg/m² X 4<br>Daunorrubicin 25mg/m² X 2<br>PEG asparaginase 2500UI/m² X 1<br>Prednisone 40mg/m² D1 to D26 (reduction until D30)<br>MADIT X 3 (4X if CNS2)<br><br>Phase 2<br>-	Phase 1<br>VCR 1.5mg/m² X 4<br>Daunorrubicin 25mg/m² X 2<br>PEG asparaginase 2500UI/m² X 1<br>Prednisone 40mg/m² D1 to D28 (reduction until D30)<br>MADIT X 3 (4X if CNSC2)<br><br>Phase 2<br>Cyclophosphamide 1000mg/m² X 1<br>Cytarabine 75mg/m² X 4 days X 2 weeks<br>6MP 60mg/m²/dia 14days<br>MADIT 1X<br>*PEG 2500 UI/m² X 1 if MDR D19 ≥ 0.1%	Phase 1<br>VCR 1.5mg/m² X 4<br>Daunorrubicin 25mg/m² X 2<br>PEG asparaginase 2500UI/m² X 1<br>Prednisone 40mg/m² D1 to D28 (reduction until D33)<br>MADIT X 3 (4X if CNS2 or CNS 3)<br><br>Phase 2<br>Cyclophosphamide 1000mg/m² X 1<br>Cytarabine 75mg/m² X 4 dias X 2 weeks<br>6MP 60mg/m²/dia 14days<br>MADIT 1X<br>*PEG 2500 UI/m² X 1 if MRD D19 ≥ 0.1%
**Evaluation Moments of MRD**	D15<br>D33<br>D78	D15<br>D33<br>D78	D15<br>D33<br>D78	D19<br>D26<br>D49<br>After consolidation (if MRD+ on D49)	D19<br>D49<br>After consolidation (if MRD+ on D49)	D19<br>D49<br>After consolidation (if MRD+ on D49)

Supplementary Frame 2. Consolidation treatment of adapted ALLIC BFM 2009 and GBLT2021 protocols

**Protocol**	BFM adapted			GBTLI 2021			
**Risk Classification**	Low Risk	Intermediate Risk (IR)	High Risk (HR)	True Low Risk	Intermediate Risk (IR)	High Risk (HR)	Extreme High Risk
**Risk Criteria (after induction)**	Age < 6yo<br>and<br>WBC < 20000/mm³<br>and<br>MDR D15 < 0.1%<br>and<br>MDR D33 < 0.01%<br>and<br>MDR D78 < 0.01%	No low risk and<br>No high-risk criteria	MRD at D15 > 5% or<br>MRD at D78 > 0.01%	PLR and<br>MDR D19 < 0.01	Previous use of corticosteroid<br>PLR and MDR D19 ≥0.01 and <5<br>and MDR D49 <0.01<br>Patients with WBC >50000/mm² and < 10yo and hyperdiploidy ou *ETV6::RUNX1*	HR at initial diagnosis<br>or<br>MDR ≥5<br>or<br>MDR D49 ≥ 0.01	MDR D49 > 5%<br>or<br>MDR pos consolidation ≥ 0.01
**Consolidation Chemotherapy**	MTX 2g/m² over 24h X 4 (4 biweekly cycles)<br>MADIT X 4<br>6MP 25mg/m² for 56 days	MTX 5g/m² over 24h X 4 (4 biweekly cycles)<br>MADIT X 4<br>6MP 25mg/m² for 56 days	Three distinct cycles, two times each cycle.<br><br>HR1<br>MTX 5g/m² over 24hours<br>Dexamethasone 20mg/m² 5d<br>Vincristine1.5mg/m² X 2<br>Cyclophosphamide 200mg/m² X 5<br>Cytarabine 2g/m²X 2<br>PEG asparaginase 2500UI/m² X1<br>MADIT X1 (2x if CNS3)<br><br>HR2<br>MTX 5g/m² in 24 hours<br>Dexamethasone 20mg/m² 5d<br>Vinblastine 6mg/m²<br>Ifosfamide 800mg/m² X 5<br>Daunorubicin 30mg/m² X1<br>PEG asparaginase 2500UI/m²<br>MADIT X1 (2x if CNS3)<br><br>HR3<br>Dexamethasone 20mg/m² 5d<br>Cytarabine 2g/m²X 4<br>Etoposide 100mg/m² X5<br>PEG asparaginase 2500UI/m²<br>MADIT X 1	MTX 2.5g/m² over 6h X 4 (4 biweekly cycles)<br>MADIT X 4<br>6MP 25mg/m² for 56 days	MTX 2.5g/m² over 6h X 4 (4 biweekly cycles)<br>MADIT X 4<br>6MP 25mg/m² for 56 days	MTX 5g/m² over 6h X 4 (4 biweekly cycles)<br>MADIT X 4<br>6MP 25mg/m² for 56 days	MTX 5g/m² over 6h X 2 (2 biweekly cycles)<br>MADIT X 2<br>6MP 25mg/m² for 28 days<br><br>If MDR negative after 2 cycles<br>Complete four cycles of MTX 5g/m² over 6h (2 biweekly cycles) and use 6MP for 56 days<br><br>If MDR positive after 2 cycles:<br><br>Dexamethasone 20mg/m² 6 days<br>Cytarabine 2g/m²X 4<br>Etoposide 100mg/m² X5<br>PEG asparaginase 2500UI/m²<br>MADIT X 1

Supplementary Frame 3 (to be continue). Re-induction and maintenance treatment of adapted ALLIC BFM 2009 and GBLT2021 protocols

**Protocol**	ALLIC 2009 BFM adapted			GBTLI 2021			
**Risk Classification**	Low Risk<br><br>(106 weeks)	Intermediate Risk (106weeks)	High Risk (HR)<br><br>(106weeks)	True Low Risk<br><br>(104 weeks)	Intermediate Risk (IR)<br><br>(104 weeks)	High Risk (HR) (104 weeks)	Extreme High Risk<br><br>(104 weeks)
**Re-induction and maintenance therapy**	Re-induction<br>(6 weeks)<br>Dexamethasone PO 21days<br>VCR 1.5mg/m² X4<br>Doxorrubicin 30mg/m² X2<br>PEG asparaginase 2500U/m² X1<br>Cyclophosphamide 1g/m² X1<br>Cytarabine 75mg/m² X 4 days X 2 weeks<br>6TG PO 14 days<br>MADIT X2 (4 if CNS3)<br><br>Maintenance<br>(76 weeks))<br>MTX PO<br>6MP PO<br>VCR + dexamethasone each 8 weeks<br>MADIT each 8 weeks (2x)	Re-induction<br>(6 weeks)<br>Dexamethasone PO 21days<br>VCR 1.5mg/m² X4<br>Doxorrubicin 30mg/m² X4<br>PEG asparaginase 2500U/m² X1<br>Cyclophosphamide 1g/m² X1<br>Cytarabine 75mg/m² X 4 days X 2 weeks<br>6TG PO 14 days<br>MADIT X2 (4 if CNS3)<br><br>Maintenance<br>(76 weeks))<br>MTX PO<br>6MP PO<br>VCR + dexamethasone each 8 weeks<br>MADIT each 8 weeks (2x)	Re-induction<br>(6 weeks)<br>Dexamethasone PO 21days<br>VCR 1.5mg/m² X4<br>Doxorrubicin 30mg/m² X4<br>PEG asparaginase 2500U/m² X1<br>Cyclophosphamide 1g/m² X1<br>Cytarabine 75mg/m² X 4 days X 2 weeks<br>6TG PO 14 days<br>MADIT X2 (4 if CNS3)<br><br>Maintenance<br>(76 weeks))<br>MTX PO<br>6MP PO<br>VCR + dexamethasone each 8 weeks<br>MADIT each 8 weeks (2x)	1st maintenance<br>(6 weeks)<br>Dexamethasone<br>VCR 1.5mg/m²X1<br>MTX IM 5 weeks<br>6-MP PO 5weeks<br>MADIT 1x<br><br>Re-induction<br>(6 weeks)<br>Dexamethasone PO 7days (X2)<br>VCR 1.5mg/m² X4<br>PEG asparaginase 2500U/m² X1<br>MTX 2.5mg/m² over 6hours X 1<br>6MP PO 14 days<br>MADIT X2 (3 if CNS2 or3)<br><br>2nd maintenance<br>(40 weeks)<br>MTX IM<br>6MP PO<br>VCR + dexamethasone each 4 weeks<br>MADIT each 8 weeks (5X)<br><br>3nd maintenance maintenance<br>MTX IM<br>6MP PO until week 104	1st maintenance<br>(6weeks)<br>Dexamethasone<br>VCR 1.5mg/m²X1<br>MTX IM 5 weeks<br>6-MP PO 5weeks<br>MADIT 1x<br><br>1st Re-induction<br>(6 weeks)<br>Dexamethasone PO 7 days (X2)<br>VCR 1.5mg/m² X4<br>PEG asparaginase 2500U/m² X1<br>MTX 2.5mg/m² over 6hours X 1<br>6MP PO 14 days<br>MADIT X2<br><br>2nd maintenance (12 weeks)<br>MTX IM<br>6MP PO<br>VCR + dexamethasone each 4 weeks<br>MADIT each 8 weeks (2x)	1st maintenance<br>(6weeks)<br>Dexamethasone<br>VCR 1.5mg/m²X1<br>Doxorrubicin 30mg/m² X1<br>PEG asparaginase 2500U/m² X1<br>MTX IM 5 weeks<br>6-MP PO 5weeks<br>MADIT 1x<br><br>1st Re-induction<br>(6 weeks)<br>Dexamethasone PO 7 days (X2)<br>VCR 1.5mg/m² X4<br>Doxorrubicin 30mg/m² X1<br>PEG asparaginase 2500U/m² X1<br>MTX 5mg/m² over 6hours X 1<br>6MP PO 14 days<br>MADIT X2<br><br>2nd maintenance (12 weeks)<br>MTX IM<br>6MP PO<br>VCR + dexamethasone each 4 weeks<br>MADIT each 8 weeks (2x)	Blinatumumab<br>Bone Marrow Transplantation

Supplementary Frame 3 (continuation). Re-induction and maintenance treatment of adapted ALLIC BFM 2009 and GBLT2021 protocols

**Protocol**	ALLIC 2009 BFM adapted			GBTLI 2021			
**Risk Classification**	Low Risk<br><br>(106 weeks)	Intermediate Risk<br>(106weeks)	High Risk (HR)<br><br>(106weeks)	True Low Risk<br><br>(104 weeks)	Intermediate Risk (IR)<br><br>(104 weeks)	High Risk (HR)<br>(104 weeks)	Extreme High Risk<br><br>(104 weeks)
**Re-induction and maintenance therapy**					2st Re-induction<br>(5 weeks)<br>Dexamethasone PO 7 days (X2)<br>VCR 1.5mg/m² X3<br>Doxorrubicin 30mg/m² X1<br>PEG asparaginase 2500U/m² X1<br>MTX 2.5mg/m² over 6hours X 1<br>6MP<br>MADIT X1<br><br>3nd maintenance<br>(23weeks)<br>MTX IM<br>6MP PO<br><br>VCR + dexamethasone each 4 weeks<br>MADIT each 8 weeks (2x)<br><br>4nd maintenance<br>MTX IM<br>(52 weeks)<br>6MP PO<br><br>VCR + dexamethasone each 4 weeks	2st Re-induction<br>(4 weeks)<br>Dexamethasone PO 7 days (X2)<br>VCR 1.5mg/m² X2<br>Doxorrubicin 30mg/m² X1<br>PEG asparaginase 2500U/m² X1<br>MTX 5mg/m² over 6hours X 1<br>6MP<br>MADIT X1<br><br>3nd maintenance<br>(24weeks)<br>MTX IM<br>6MP PO<br><br>VCR + dexamethasone each 4 weeks<br>MADIT each 4 weeks (6x)<br><br>4nd maintenance<br>(52 weeks)<br>MTX IM<br>6MP PO<br><br>VCR + dexamethasone each 4 weeks	

Supplementary Table 1. Clinical Characteristics and Evolution of patients with B-ALL (n=156)

		ALL PATIENTS (156)	BFM protocol (n=122)	GBTLI protocol (n=34)
Age (months)	Minimum	14m	16m	14m
	Maximum	212m	212m	209m
	Median	53m	51m	60m
Sex	Male:	80 (51%)	67 (54.9%)	13 (38%)
	Female:	76 (49%)	55 (45.1%)	21 (62%)
	Relationship ♂/♀	1.05	1.21	0.61
Leukocyte count (mm³)	Minimum	510	510	560
	Maximum	481000	481000	10995
	Median:	8135	7630	19780
CNS Status	CNS 1	94 (60.2%)	75 (61.4%)	19 (55.8%)
	CNS 2	57 (36.5%)	44 (36%)	13 (38.2%)
	CNS 3	5 (3.2%)	3 (2.45%)	2 (5.8%)
Evolution	Remission	122 (78.2%)	90 (73.7%)	32 (94.1%)
	Relapse	12 (7.7%)	11* (9.0%) (*5 óbitos)	1* (2.9%) (*progressed to death)
	Death	24 (15.4%) (*6 after relapse)	22 (18.0%) (*5 after relapse)	2 (5.9%) (*1 after relapse)
	Transferred	4	4	0
Current situation	1th Remission (finished treatment)	79	77	2
	1th Remission (ongoing treatment)	43	13	30
	2th Remission (finished treatment)	4	4	0
	Death without remission documentation	4	4	0
	Death in remission	14	13	1
	Death with active leukemia (relapse or inductory failure)	6	5	1
	Relapses in treatment (remission)	2	2	0
	Loss of follow up	4	4	0

Supplementary Table 2. Characteristics of patients who presented recurrence

P	Age (years)	Gender	WBC	CNS	Risk Group	Treatment Protocol	MRD D15/D19*	MRD D78/49	Pos Induction Risk Group	Site of relapse	Time to relapse	Situação Atual	GS (m)	Biological Marker	FLT3 exp1 RQ	FLT3 Exp2 RQ
21	1	M	25880	CNS 1	RI	BFM ALLIC 2009 adap	0.18	NR	IR	testicles	39	ROT	64	*ETV6::RUNX1*	0.17	0.07
23	12	M	308000	CNS 1	AR	BFM ALLIC 2009 adap	11	0.04	HR	BM	3	DL	9	Hypodiploidy	1.76	0.26
27	2	M	38500	CNS 2	RI	BFM ALLIC 2009 adap	0.34	NR	IR	CNS	15	ROT	65	*TCF3::PBX1* Low Hiperdiploidy	0.09	0.12
51	8	M	3630	CNS 1	RI	BFM ALLIC 2009 adap	0.24	<0.01	IR	CNS	25	DL	58	High Hiperdiploidy + Del *ERG* + Del *PAX5* + Del JAK2	1.39	0.10
120	11	M	1750	CNS 1	RI	BFM ALLIC 2009 adap	0.04	<0.01	IR	BM	23	RIT	55	Mut *FLT3* TKD	NA	4.5
55	2	F	8650	CNS 2	BR	BFM ALLIC 2009 adap	0.82	0.63	HR	medular	4	DL	8	Mut *FLT3* TKD	2.53	0.38
56	4	F	3530	CNS 2	BR	BFM ALLIC 2009 adap	5.21	<0.01	HR	BM	22	ROT (BMT)	49	High Hiperdiploidy	1.50	1.27
68	14	F	6300	CNS 3	RI	BFM ALLIC 2009 adap	0.34	<0.01	IR	CNS	26	OR	28	*IKZF1*^{plus}	1.15	0.07
70	2	F	6700	CNS 2	BR	BFM ALLIC 2009 adap	0.11	<0.01	IR	BM	23	ROT (BMT)	42	*ETV6::RUNX1*	0.91	0.89
125	11	M	71130	CNS 1	RI	BFM ALLIC 2009 adap	1.42	76	HR	BM	3	DL	5	*IKZF1*^{plus}	NR	1.73
76	5	M	15680	CNS 1	BR	BFM ALLIC 2009 adap	5.15	0	HR	BM	35	RIT	40	*ETV6::RUNX1*	0.32	NA
153	3	F	4160	CNS 3	AR	GBTLI 2021	3.77	0.19	RH	BM / CNS	2	DL (relapse)	7	High Hiperdiploidy	NA	NA

CNS: central nervous system; WBC: white blood cells; MRD: minimal residual disease; IR: intermediate risk; LR: Low risk; HR: High risk; Rel.: relapse; ROT: remission out of treatment; DL: death for leukemia; RIT: remission in treatment; BMT: bone marrow transplantation
