# Supplementary figures and images for: Prevalence of FLT3 gene mutation and its expression in Brazilian pediatric B-ALL patients: clinical implications
Source: Front Pediatr. 2024 Dec 6;12:1505060. doi: 10.3389/fped.2024.1505060 (PMC11658997; doi:10.3389/fped.2024.1505060)

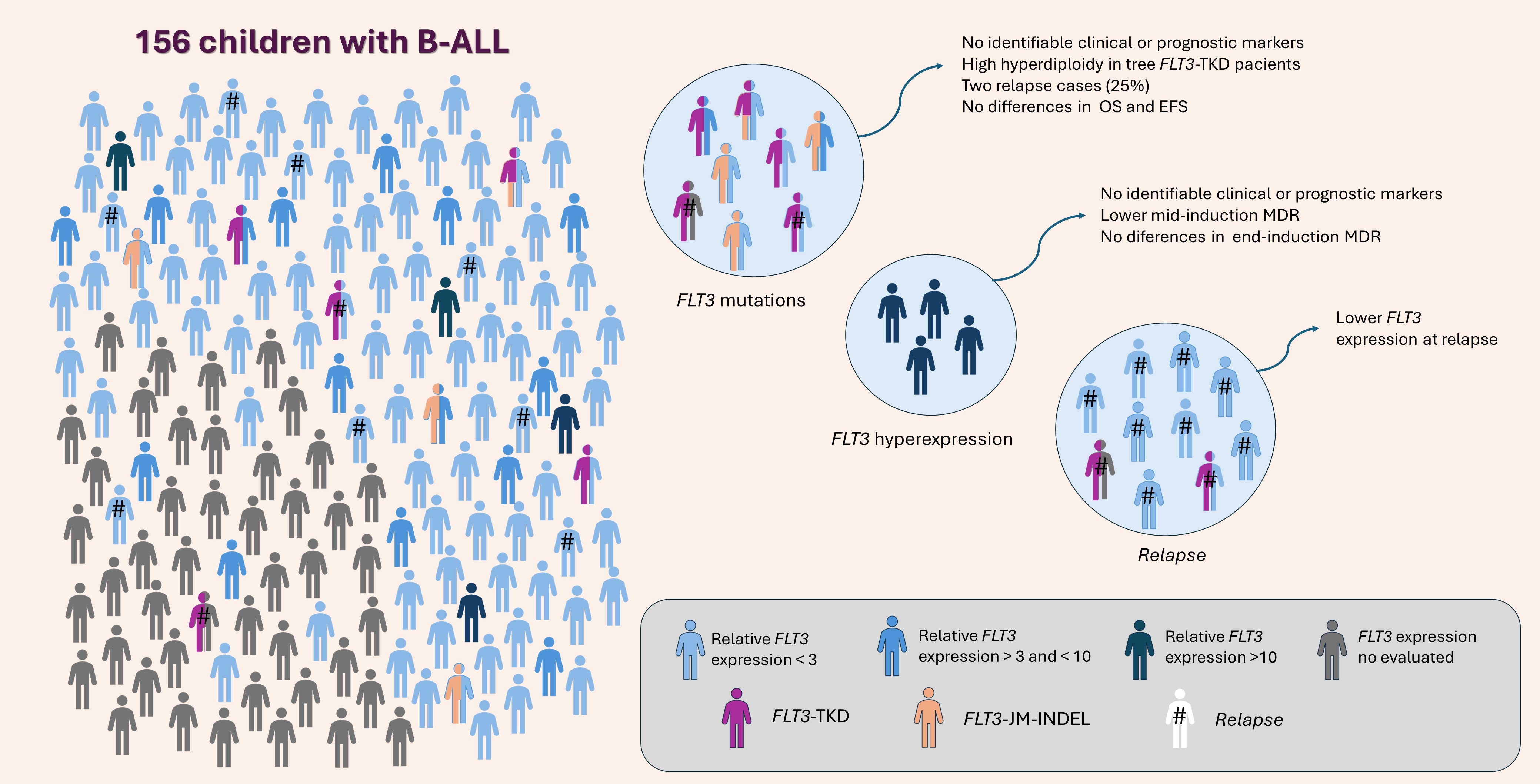

Supplement: Supplementary file 2 [file Image1.jpg]
